# Evaluation of the Immunity Responses in Mice to Recombinant *Bacillus subtilis* Displaying Newcastle Disease Virus *HN* Protein Truncations

**DOI:** 10.3390/microorganisms12030439

**Published:** 2024-02-21

**Authors:** Jianzhen Li, Miao Yang, Bin Chen, Zhenhua Wang, Yuheng Cao, Yang Yang, Mengwei Zhang, Dongmei Zhang, Xueqin Ni, Yan Zeng, Kangcheng Pan

**Affiliations:** 1 Animal Microecology Institute, Department of Animal and Plant Quarantine, College of Veterinary Medicine, Sichuan Agricultural University, Chengdu 611130, China; 2 College of Animal Husbandry and Veterinary, Chengdu Agricultural College, Chengdu 611130, China; 3 Technology Center of Chengdu Customs, Chengdu 610041, China

**Keywords:** *Bacillus subtilis*, Newcastle disease virus (NDV), spore surface display, mucosal immunity

## Abstract

*Bacillus subtilis*, a probiotic bacterium with engineering potential, is widely used for the expression of exogenous proteins. In this study, we utilized the integrative plasmid pDG364 to integrate the hemagglutinin–neuraminidase (*HN*) gene from Newcastle disease virus (NDV) into the genome of the *B. subtilis* 168 model strain. We successfully constructed a recombinant *B. subtilis* strain (designated *B. subtilis* RH) that displays a truncated *HN* antigen fragment on the surface of its spores and further evaluated its immunogenic effects in mice. Using ELISA, we quantified the levels of IgG in serum and secretory IgA (sIgA) in intestinal contents. The results revealed that the recombinant *B. subtilis* RH elicited robust specific mucosal and humoral immune responses in mice. Furthermore, *B. subtilis* RH demonstrated potential mucosal immune adjuvant properties by fostering the development of immune organs and augmenting the number of lymphocytes in the small intestinal villi. Additionally, the strain significantly upregulated the relative expression of inflammatory cytokines such as IL-1β, IL-6, IL-10, TNF-α, and IFN-γ in the small intestinal mucosa. In conclusion, the *B. subtilis* RH strain developed in this study exhibits promising mucosal immunogenic effects. It holds potential as a candidate for an anti-NDV mucosal subunit vaccine and offers a novel preventive strategy for the poultry industry against this disease.

## 1. Introduction

*Bacillus subtilis* is a probiotic bacterium that is widely used for engineering purposes to express exogenous proteins [1,2]. Under conditions of extreme stress or nutrient scarcity, *B. subtilis* generates a distinct cellular form known as the spore. The spore’s core and cortex are crucial for its formation and integrity. Encompassing the cortex is the spore’s coat layer, composed of approximately 80 unique coat proteins, which collectively define the spore surface [3]. This robust architecture endows *B. subtilis* spores with high resistance, enabling them to withstand acid and bile salt challenges within the intestine tract [4,5]. Beyond its capacity to elicit both mucosal and humoral immune responses within the host, *B. subtilis* also functions as an effective carrier or adjuvant for mucosal vaccine delivery [6]. Leveraging spore surface display technology enables the stable presentation of antigenic proteins on the spore surface. Research efforts have yielded the development of protective antigens through the utilization of diverse coat proteins, such as *cotB*, *cotC*, *cotG*, etc., as anchoring agents. This approach facilitates the expression of antigenic proteins on the spore surface and culminates in the creation of recombinant probiotics that are rich in immunogenic properties [7,8,9]. Recombinant strains of *B. subtilis* are characterized by their ease of storage and transport, which simplifies the immunization process and reduces stress in animals. These strains exhibit a broad spectrum of applications in the field of animal immunization. Recent advances have led to the successful display of various antigenic proteins on the spore surface, including tetanus toxoid fragment C, ovalbumin [10,11], cholera toxin [12], the transmissible gastroenteritis virus spike protein [13], the highly pathogenic avian influenza H5N1 hemagglutinin protein [14], and the *Vibrio* OmpK protein [15]. The induction of specific immune responses by these recombinant probiotics underscores their exceptional immune attributes and probiotic functionalities.

Newcastle disease virus (NDV) is the causative agent of Newcastle disease (ND), a significant poultry disease. Characterized by high contagiousness, ND is categorized by the World Organization for Animal Health (WOAH) as a List A disease in animals, necessitating legal reporting [16]. In China, the Ministry of Agriculture ranks it as a Class Ⅱ animal epidemic disease. Challenges in eradicating NDV stem from suboptimal vaccination, antigenic drift, limited duration of immunity, and immunosuppressive conditions [17,18,19]. The hemagglutinin–neuraminidase (*HN*) protein, a surface glycoprotein of NDV, is renowned for its ability to elicit the production of neutralizing antibodies within poultry, thereby serving as a pivotal protective antigen. The globular head domain of the *HN* protein houses the essential functional regions, including the active site and all antigenic sites [20,21]. This domain is instrumental in the virus’s infectivity and pathogenicity, positioning the *HN* protein as an optimal target for the genetic engineering of vaccines aimed at combating ND [22,23,24]. Currently, the main types of genetically engineered vaccines for ND include nucleic acid vaccines, subunit vaccines, and live-vector vaccines [25,26,27]. The attachment and entry of NDV into host cells are facilitated by its binding to two major salic acid (SA) receptors present on the membrane of target cells. Specifically, SAα2,3-Gal receptors are prevalent in chicken small intestinal epithelial cells, while SAα2,6-Gal receptors are more common in avian trachea ciliated epithelial cells [28,29]. Consequently, NDV exhibits a specific mucosal tropism and can readily infect birds through the respiratory and digestive tracts. Mucosal vaccines have the capacity to elicit the production of sIgA, establishing mucosal immunity through various immunization routes such as intranasal, ocular, drinking water, and aerosol administration. These methods significantly enhance local immunity within the respiratory and digestive tracts [30,31]. In summary, mucosal immunization plays a crucial role in managing ND, and the development of recombinant probiotic-based mucosal vaccines against NDV is currently a paramount strategy in this field.

In this study, we engineered probiotics using the *B. subtilis* model strain 168. By integrating a fusion gene into the genome of *B. subtilis* 168 via the integration plasmid pDG364, we successfully constructed the recombinant *B. subtilis* RH. This strain displays the truncated *HN* (*HNJD*) protein of Newcastle disease virus on its spore surface. We then proceeded to assess the specific immune response and mucosal immune adjuvant effect elicited by *B. subtilis* RH in mice. The novel oral vaccine developed herein offers an innovative approach for the prevention of NDV-related diseases, presenting a fresh alternative in disease management strategies.

## 2. Materials and Methods

### 2.1. Strains, Vaccines, Cell, Plasmids, Primers Sequences, and Experimental Ethics

The bacterial strains (*Escherichia coli* BL21/DH5α and *Bacillus subtilis* 168), vaccines (NDV vaccine strain LaSota/CS2), chick embryo fibroblasts (CEF), plasmids (pUCm-T, pET-32a, and pDG364-*cotB*), and primer sequences utilized in this study are itemized in Supplementary Table S1. All methodologies and animal experimentation were carried out in accordance with the “Guide for the Care and Use of Laboratory Animals”, receiving approval from the Institutional Animal Care and Use Committee of Sichuan Agricultural University (approval number: SYXK Chuan 2019-187).

### 2.2. Cloning of Truncated HN Gene

The E.Z.N.A^®^ Total RNA Kit II (Omega, Honolulu, HI, USA) was used to extract the total viral RNA from the NDV LaSota vaccine strain (GenBank accession No. AY845400.2). Subsequently, the extracted RNA was reverse-transcribed to synthesize cDNA. The hemagglutinin–neuraminidase-truncated (*HNJD*) gene was PCR-amplified using the cDNA as a template and *HNJD*-F1/R1 primers [32]. The amplicon was then T-A-cloned into a pUCm-T vector to generate the recombinant plasmid pUCm-T-*HNJD*, which was sequenced and subjected to site-directed mutagenesis to eliminate potential restriction sites that might interfere with the subsequent steps (performed by Tsingke Biotechnology Co., Ltd., Beijing, China).

### 2.3. Preparation of Hyperimmune Serum and Prokaryotic Expression of HNJD

Female New Zealand rabbits weighing 2.0 ± 0.2 kg were used to produce anti-NDV hyperimmune serum. Each rabbit received a subcutaneous injection of 2 mL of the live vaccine (LaSota strain). This was followed by weekly booster immunizations, and serum collection occurred subsequent to three consecutive vaccinations. Hemagglutination inhibition (HI) assays were performed to determine the HI antibody titers against NDV, and optimal dilutions were ascertained and preserved for further use.

The pUCm-T-*HNJD* plasmid and the pET-32a vector were digested with *BamH* I and *EcoR* I enzymes (Takara Bio, Dalian, China). The resulting target fragments were ligated to form the expression plasmid pET-32a-*HNJD*1, which was then transformed into *E. coli* BL21. The successfully expressing strain was designated as *E. coli* BL21/pET-32a-*HNJD*. The expression of the recombinant protein was induced with isopropyl-β-D-thiogalactoside (IPTG) added to a Luria–Bertani (LB) broth at a final concentration of 1 mM. After incubation at 37 °C with shaking at 160 rpm for 6 h, 1 mL of bacterial culture was collected, while the pET-32a vector alone served as a negative control. The bacterial cells were lysed by ultrasonication and centrifuged at 10,000× *g* for 1 min. The pellet was collected and subjected to SDS-PAGE, followed by transfer onto a PVDF membrane (Solarbio, Beijing, China). The membrane was blocked with 5% skim milk at 37 °C for 2 h and then incubated overnight at 4 °C with rabbit-derived NDV hyperimmune serum (diluted 1:200 in TBST). HRP-conjugated goat anti-rabbit IgG (diluted 1:2000 in TBST, BOSTER, Wuhan, China) was used as the secondary antibody. After a 2 h incubation at room temperature and washing with TBST, color development was performed using the DAB Color Development Kit (BOSTER, Wuhan, China) according to the manufacturer’s instructions. If the *HNJD* protein specifically binds to anti-NDV hyperimmune serum, a brown band should be visible on the membrane after immunoblotting.

### 2.4. Construction and Transformation of Recombinant Integration Plasmid

**Figure 1:**
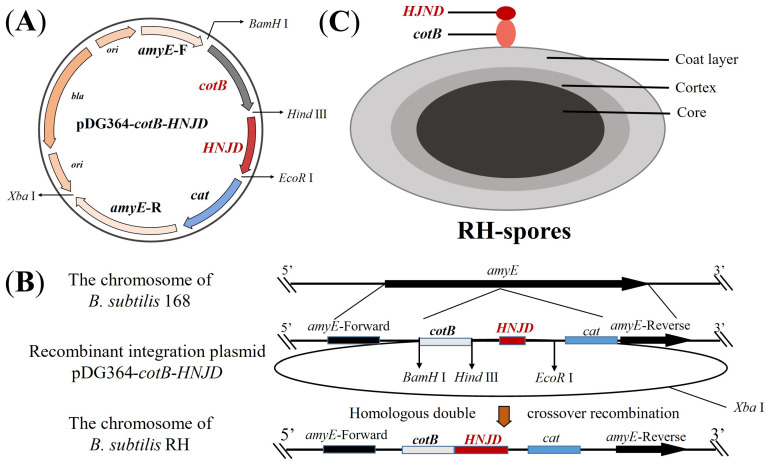
Schematic representation of homologous recombination and the structure of RH spores. (**A**) Diagram of the integrative plasmid pDG364-*cotB*-*HNJD*. (**B**) Homologous double-crossover recombination between the recombinant plasmid pDG364-*cotB*-*HNJD* and the genome of *B. subtilis* 168 occurs, leading to the integration of the target gene into the *amyE* locus. (**C**) Structure depiction of RH spores showing the fusion expression of the anchor protein *cotB* and the *HNJD* protein on the surface of the spores.

The plasmid pDG364-*cotB* developed in our previous work [33], was utilized. Employing the methodology detailed in Section 2.3, we constructed the recombinant plasmid pDG364-*cotB*-*HNJD*. Competent cells of *B. subtilis* 168 were prepared following Julkowska et al.’s protocol [34]. The plasmid pDG364-*cotB*-*HNJD* was linearized with an *Xba* I enzyme digestion prior to being introduced into the competent cells. Through homologous double-crossover recombination, the target gene was integrated into the *amyE* (amylase) locus of *B. subtilis* (Figure 1B). Positive clones were identified on chloramphenicol (5 μg/mL) resistance plates and further screened on a 1% starch nutrient agar medium. Genomic DNA from the recombinant bacteria was extracted, and a PCR analysis was performed with primer pairs *amyE*-F/R, *amyE*-F/*HNJD*-R2, *cotB*-F/*HNJD*-R2, and *HNJD*-F2/R2. Amplification products were verified by gel electrophoresis. The correctly identified recombinant strain was designated *B. subtilis* RH.

### 2.5. Sporulation and Immunofluorescence Microscopy

Sporulation was induced using Difco sporulation medium (DSM) according to the method described by Stasiłojć et al. [35]. After approximately 24 h of sporulating culture, spores were treated with 2.0 mg/L of lysozyme at 37 °C for 2 h to eliminate any residual vegetative cells. The purified spores were then obtained by sequential washing with 1 M of NaCl, 1 M of MgCl_2_, and distilled water, as detailed in reference [7]. Finally, the spread plate technique was employed to enumerate the spore colonies.

Immunofluorescence microscopy was used to verify the successful display of the *HNJD* protein on the spore surface [36], as indicated in Figure 1C. A purified spore suspension was prepared and affixed to microscope slides according to the method described previously [37]. Rabbit anti-NDV hyperimmune serum (diluted 1:200 in PBST) served as the primary antibody, while Cy3-conjugated goat anti-rabbit IgG (diluted 1:200 in PBST, BOSTER, Wuhan, China) was used as the secondary antibody. Additionally, serum from non-immunized mice was applied as the primary antibody to establish a negative control. The immunofluorescence images were captured using a fluorescence microscope (DMi8, Leica, Tokyo, Japan).

### 2.6. Immunization of Mice and Collection of Samples

**Figure 2:**
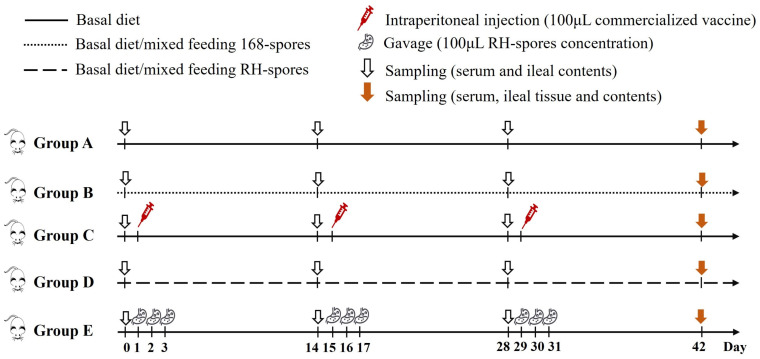
Schematic representation of the vaccination and feeding trail. Group A: untreated control; Group B: mixed diet with 168-spores; Group C: inactivated vaccine administered; Group D: mixed diet with RH-spores; Group E: RH-spores administered via gavage.

A total of 100 three-week-old female BALB/c mice underwent a seven-day acclimatization period before being randomly distributed into five groups, each comprising 20 individuals. Each group was then subdivided into five cages, with four mice per cage. The feeding regimen and immunization schedule for the mice are detailed in Figure 2. The mice in group A served as the control group and were provided with a basal diet. Group B received a diet mixed with 2.0 × 10^6^ CFU per gram of wild-type *B. subtilis* 168 spores (168-spores). Group C was intraperitoneally administered 100 μL of inactivated vaccine (strain LaSota, with viral content equal to or exceeding 1.0 × 10^8^ ELD50 prior to inactivation) on days 1, 15, and 29. Group D was fed a diet containing 2.0 × 10^6^ CFU per gram of *B. subtilis* RH spores (RH-spores). Group E received an RH-spore suspension (2.0 × 10^10^ CFU/mL) via oral gavage. The mice in group E received 0.1 mL daily on days 1–3, 15–17, and 29–31, as previously described [38]. Each mouse was given 3–5 g of this diet daily.

The experimental period spanned 42 days. On days 0, 14, 28, and 42, five mice from each group were randomly selected, and their serum and small intestinal contents were harvested and preserved at −80 °C. The body weight of the mice was recorded on day 42. Following euthanasia, ileal tissues and contents were gathered, and the ileal tissues were preserved in 4% paraformaldehyde. The spleens and thymuses were excised, and organ indices were calculated as follows:

organ index=weight of organ (mg)/weight of body (g)

### 2.7. Detection of NDV-Specific Serum and Mucosal Antibodies

Serum IgG antibody levels were quantified using a competitive enzyme-linked immunosorbent assay (ELISA) with the Serum Antibodies to Newcastle Disease Virus ELISA Kit (Zhenrui Bio, Shenzhen, China). The serum IgG levels were expressed as *S*/*N* (sample-to-negative control) values:

S/N=(OD450 nm of sample well)/(OD450 nm of negative control well)

The indirect ELISA method was used to detect the anti-NDV sIgA in the small intestinal contents. The ELISA plates were coated with NDV antigen, provided by Shenzhen Zhenrui Biotech Co., Ltd., Shenzhen, China. The intestinal contents were diluted to 1:100 with PBST, and serum from unimmunized mice served as the negative control. The secondary antibody used was horseradish peroxidase (HRP)-conjugated goat anti-mouse IgA (diluted to 1:1200 in PBST, Abcam, Cambridge, UK). The levels of sIgA in the small intestinal contents were expressed as *P*/*N* (positive-to-negative control) values:

P/N=(OD450 nm of sample well)⁄(OD450 nm of negative control well)

### 2.8. Microneutralization Test

The serum-neutralizing antibody (NA) titers were detected with a microneutralization assay, as previously described [39]. CEF cells were seeded in 96-well plates and used for virus neutralization tests when they reached a confluency rate of 50–60%. All serum samples underwent heat inactivation at 56 °C for 30 min and were then serially diluted two-fold from 1:50 to 1:1600 in Dulbecco’s Modified Eagle Medium (DMEM). Subsequently, 50 μL of the NDV CS2 vaccine strain (200 TCID50) was combined with an equal volume of the diluted serum and incubated together at 37 °C for 1 h. This mixture was then added to a 96-well plate containing CEF cells, with three replicate wells for each sample. The cells were maintained in DMEM supplemented with 2% fetal bovine serum (FBS) at 37 °C and 5% CO_2_. It is crucial to note that if the virus is neutralized by serum antibodies, it will be unable to infect the cells. After incubation for 72 h at 37 °C and 5% CO_2_, the NA titer for each group was calculated as the geometric mean titer (GMT), which was established by identifying the lowest serum dilution that prevented cytopathic effects in over 50% of the CEF cells.

### 2.9. Hemagglutination Inhibition Test

The HI assay was performed in accordance with the National Standard of the People’s Republic of China [40]. Serum samples were twofold diluted in a 96-well plate. A total of 25 μL of NDV antigen containing four hemagglutination units was introduced into each well, including a positive control. After 30 min of incubation, 25 μL of a 1% suspension of chicken red blood cells was added to each well and incubated for an additional 30 min. The results were observed, and the HI titers were defined as the highest dilution of serum that completely inhibited NDV hemagglutination.

### 2.10. Gene Expression of Cytokine in Ileum

Total RNA from ileal tissues samples was extracted using the Animal Total Isolation Kit (Foregene Co., Ltd., Chengdu, China). The extracted RNA was subsequently reverse-transcribed into cDNA using the RT-Easy™ II (Foregene, Chengdu, China). The expression levels of cytokine-related mRNAs, including interleukin 1 beta (IL-1β), interleukin 6 (IL-6), interleukin 10 (IL-10), interferon gamma (IFN-γ), and tumor necrosis factor alpha (TNF-α), were quantified by real-time quantitative PCR (RT-qPCR) according to the methodology described by Xin et al. [41]. Primer sequences are listed in Supplementary Table S2. Beta-actin (β-actin) served as the reference gene for data normalization. The relative expression of the target genes was calculate using the 2^−ΔΔCT^ method:

$$\Delta \Delta CT=\left(CT,\;target\;gene-CT,\beta -actin\right)\;experimental\;group-\left(CT,target\;gene-CT,\beta -actin\right)\;control\;group$$

### 2.11. The Morphology of Ileum

Tissue sections were prepared from ileal tissue fixed in 4% paraformaldehyde (ServiceBio Technology Co., Ltd., Wuhan, China). The imaging of the target areas was performed using an Eclipse Ci-L photographic microscope (Nikon, Tokyo, Japan), following the method described previously [42]. Image analysis was conducted using Image-Pro Plus 6.0 software, with results reported in millimeters. For each intestinal sample, five random fields were selected to measure the villus height (VH) and crypt depth (CD) and to determine the VH/CD ratio. A 100× magnification field was used, and five intact villi were chosen from each tissue section for measurement. The height of the intestinal villi and the number of intraepithelial lymphocytes (IELs) were measured. The number of IELs per unit length of villus was then calculated as follows:

Number of IELs per unit height=IELs/height of intestinal villus

### 2.12. Statistical Analysis

Data were analyzed using one-way ANOVA followed by the Friedman test for multiple comparisons. Statistical analyses were conducted with IBM SPSS Statistics 26 (IBM Corporation, Armonk, NY, USA), and results are presented as mean ± standard deviation (SD). Statistical significance was denoted by letters, where different letters indicate significant differences (*p* < 0.05) between groups. Similar letters indicate no significant difference among groups (*p* > 0.05). Data visualization was performed using Origin 2021 (OriginLab, Northampton, MA, USA).

## 3. Results

### 3.1. Construction and Transformation of Recombinant Plasmid

**Figure 3:**
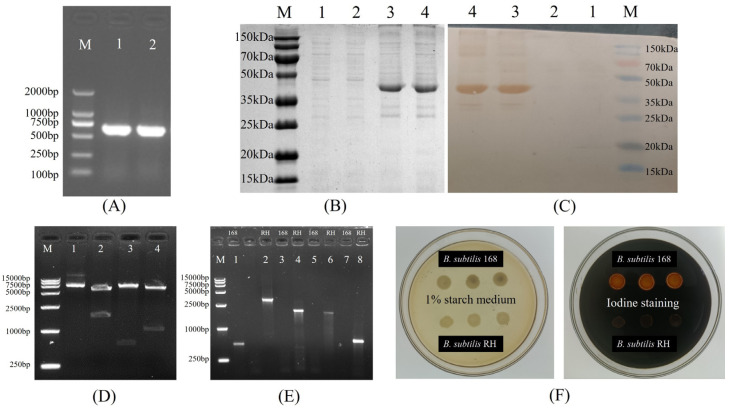
Construction of *B. subtilis* RH. (**A**) Cloning of the truncated *HNJD* gene. M: DNA marker (100–2000 bp); Lanes 1 and 2: PCR products of the *HNJD* gene. (**B**) SDS-PAGE analysis of protein expression on a 12% gel. M: Protein marker (15–150 kDa); Lanes 1 and 2: proteins expressed with the empty pET32a plasmid vector; Lanes 3 and 4: expression products after induction with IPTG for 6 h. (**C**) Western blot analysis of transmembrane proteins. (**D**) Double digestion verification of pDG364-*cotB*-*HNJD*. M: DNA marker (250–15,000 bp); Lanes 1–4: products from the recombinant plasmid digested with *Xba* I, *BamH* I/*EcoR* I, *Hind* III/*EcoR* I, and *BamH* I/*Hind* III, respectively. (**E**) PCR identification of *B. subtilis* 168 and *B. subtilis* RH. M: DNA marker (250–10,000 bp); Lanes 1 and 2: *amyE*-F/R; Lanes 3 and 4: *amyE*-F/*HNJD*-R1; Lanes 5 and 6: *cotB*-F/*HNJD*-R1; Lanes 7 and 8: *HNJD*-F2/R2. (**F**) Identification of *B. subtilis* RH with amylase activity assay. *B. subtilis* RH was cultivated on a medium containing 1% starch and then stained with iodine solution.

Figure 3 illustrates the construction of recombinant plasmids and the outcomes of the transformation. The *HNJD* gene was approximately 642 bp in size (Figure 3A). Sequencing revealed that the *HNJD* gene contained an *EcoR* I restriction site, with the original and mutated sequences detailed in Supplementary Table S3. The HI titer of the rabbit anti-NDV hyperimmune serum reached a value of 13log2. An analysis via 12% SDS-PAGE and Western blotting (shown in Figure 3B,C) confirmed that the *E. coli* BL21/pET-32a-*HNJD* expression product exhibited a protein band around 42 kDa, signifying successful recombinant protein expression with reactivity to NDV-specific antibodies. Following double-enzyme digestion validation, the recombinant plasmid pDG364-*cotB*-*NHJD* was introduced into *B. subtilis*-competent cells (Figure 3D). PCR identification was performed using four primer sets, with the wild-type *B. subtilis* genome serving as the control. The results presented in Figure 3E demonstrated that all DNA bands were congruent with their theoretical sizes. Furthermore, Figure 3F indicated that the recombinant *B. subtilis* lost its ability to break down starch due to the insertion of the fusion gene *cotB*-*HNJD* into the amylase-encoding locus. In conclusion, we successfully engineered a strain of *B. subtilis* RH.

### 3.2. Sporulation and Immunofluorescence Microscopy

**Figure 4:**
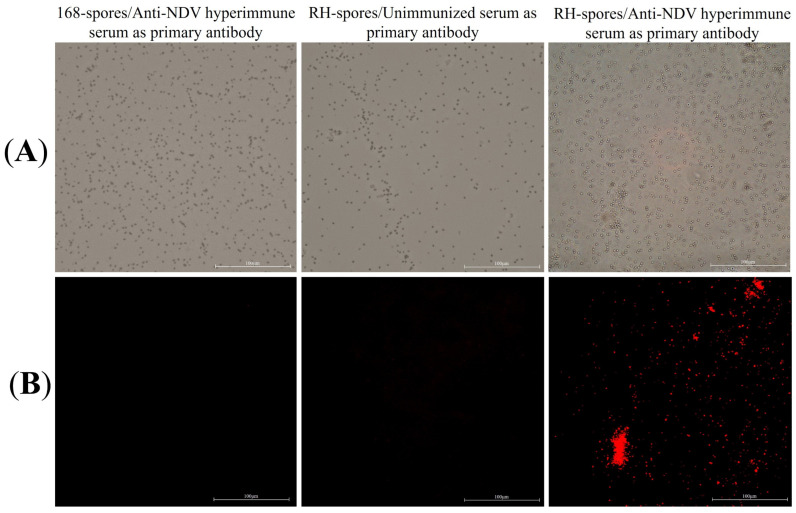
Indirect immunofluorescence assay. (**A**) Spores of *B. subtilis* 168 and RH observed under 40× magnification using bright-field microscopy; (**B**) Spores of *B. subtilis* 168 and RH under 40× magnification using fluorescence microscopy, with different serums serving as the primary antibody and Cy3-conjugated goat anti-rabbit IgG employed as the secondary antibody.

The results of the indirect immunofluorescence assay for the recombinant *B. subtilis* RH are presented in Figure 4. Red fluorescent signals were exclusively observed in *B. subtilis* RH when viewed under a microscope, using anti-NDV hyperimmune serum as the primary antibody. This observation confirms that the *HNJD* protein was successfully expressed on the surface of *B. subtilis* RH spores and was capable of binding to NDV antibodies.

### 3.3. Detection of NDV-Specific Serum and Mucosal Antibodies

**Figure 5:**
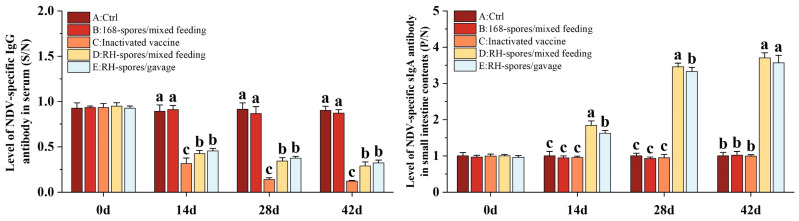
Immune responses in mice following immunization using different methods. Serum IgG levels are depicted as *S*/*N* values, while sIgA levels in the mucosa are represented by *P*/*N* values. The data are reported as mean values with SD, based on a sample size of *n* = 5. Statistical analysis involved one-way ANOVA, followed by a Friedman test. Bars labeled with distinct lowercase letters indicate a significant difference at the *p* < 0.05 level.

The ELISA results for serum IgG and mucosal sIgA are depicted in Figure 5. The serum IgG levels were quantified using a competitive ELISA approach. The *S*/*N* value is inversely related to the concentration of IgG, with lower *S*/*N* values indicating higher IgG concentrations. Throughout the study, Groups A and B exhibited consistently lower IgG levels, whereas Groups C, D, and E had significantly elevated serum IgG levels (*p* < 0.05), with Group C demonstrating the highest concentration of serum IgG. The serum IgG level was higher in Group D compared to Group E. The sIgA levels in the ileal content were determined using an indirect ELISA method. Higher *P*/*N* values are indicative of increased sIgA levels. It was observed that Groups A, B, and C did not generate substantial levels of sIgA antibodies. In contrast, both Groups D and E showed a marked increase in sIgA levels (*p* < 0.05), which progressively rose over time, reaching a plateau on day 28. Notably, Group D achieved higher levels of sIgA than Group E. This suggests that *B. subtilis* RH effectively triggered an immune response in the mice through both mixed feeding and gavage administration methods. The mixed feeding method elicited a stronger mucosal immune response, potentially due to an extended period of stimulation of the mucous membranes.

### 3.4. Detection of NA and HI Antibody Titers

**Figure 6:**
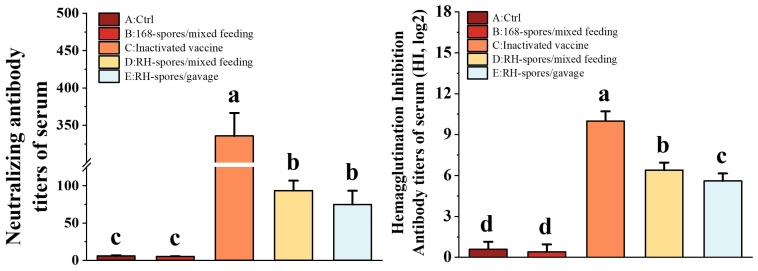
Determination of NA and HI levels in the serum of each group. The data are presented as mean ± SD, with a sample size of *n* = 5. Statistical analysis was conducted using one-way ANOVA, followed by a Friedman test. Bars labeled with different lowercase letters indicate a statistically significant difference at the *p* < 0.05 level.

Using the Reed–Muench method, the TCID50 for the NDV CS2 vaccine strain was determined to be 10^−6.2^/0.1 mL. As depicted in Figure 6, Groups C, D, and E exhibited significantly elevated NA and HI titers compared to Groups A and B (*p* < 0.05). Group C achieved the highest NA and HI titers, and Group D exhibited significantly higher HI titers than Group E (*p* < 0.05). These findings indicate that mucosal immunization can elicit the robust production of neutralizing antibodies in mice and that the levels of NA and HI antibodies are correlated with the route of mucosal administration.

### 3.5. Gene Expression of Cytokine in the Ileum

**Figure 7:**
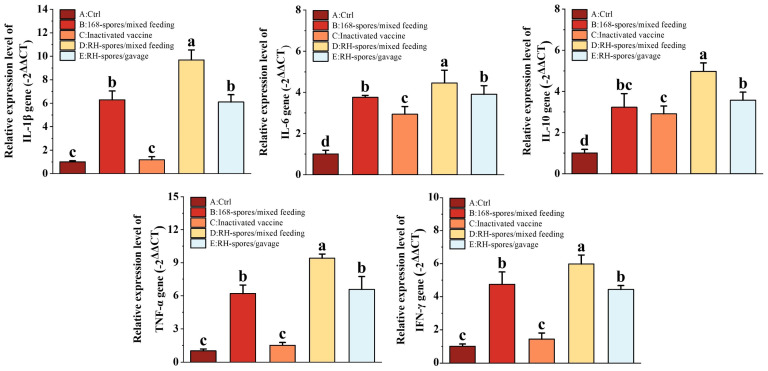
Expression levels of cytokines IL-1β, IL-6, IL-10, TNF-α, and IFN-γ in the small intestine were quantified using RT-qPCR. The data were normalized using the −2^ΔΔCT^ method. The results are presented as mean ± SD, with a sample size of *n* = 5. Statistical analysis was conducted using one-way ANOVA, followed by a Friedman test. Bars labeled with distinct lowercase letters denote a statistically significant difference at the *p* < 0.05 level.

Cytokines related to immunity were quantified using RT-qPCR, and the results are shown in Figure 7. We observed a significant upregulation in the relative mRNA expression of IL-1β, IL-6, IL-10, TNF-α, and IFN-γ in the spore-immunized groups (Group B, D, and E). Notably, all cytokine mRNA expression levels were higher in Group D compared to Group E (*p* < 0.05), indicating that mixed feeding was more effective than gavage administration. While the expression of IL-6 and IL-10 was significantly elevated in Group C compared to Group A (*p* < 0.05), their levels remained lower than those in the spore-immunized groups. This indicates that inactivated vaccines can enhance cytokine expression in the intestinal mucosa by modulating the systemic immune response, but this effect is less pronounced than the direct action of the spores on the mucosal surface.

### 3.6. Histomorphology and Intraepithelial Lymphocytes of the Mouse Ileum

**Figure 8:**
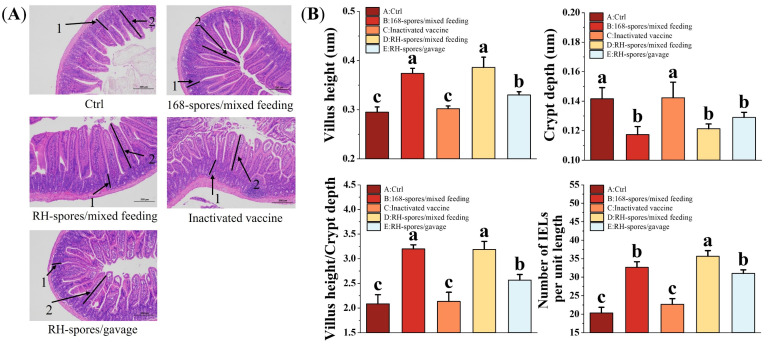
Histomorphologic analysis and enumeration of IELs in ileal tissue. (**A**) Representative images of ileal tissue from each group are shown, with intestinal crypt (1) and villi (2) indicated by black arrows. IELs are localized within the mucosal epithelial layer of the small intestine, characterized by their irregularly shaped nuclei and darker staining relative to the surrounding epithelium. Microscopic magnification is set at 100×. (**B**) Quantitative measurements of intestinal CD, VH, VH/CD ratio, and IELs per unit length are presented. Data are presented as the mean ± SD, with a sample size of *n* = 5. Statistical analysis involved one-way ANOVA, followed by a Friedman test, where bars labeled with distinct lowercase letters signify a significant difference at the *p* < 0.05 level. VH refers to the height of the villus, CD denotes the depth of the crypts, and the VH/CD ratio represents the metric of the villus height relative to the crypt depth. IELs are defined as intestinal intraepithelial lymphocytes.

Figure 8A illustrates the ileum morphology of the mice from each group, revealing a clear and intact histological structure of the ileum. The measurements of VH and CD in the mouse ileum are shown in Figure 8B. The mice from the spore-immunized groups exhibited a significant increase in VH and the VH/CD ratio and a decrease in CD compared to Groups A and C (*p* < 0.05). However, no significant differences were observed among the spore-immunized groups (*p* > 0.05). The number of IELs per unit length of the intestinal villi was quantified and is also presented in Figure 8B. The spore-immunized groups exhibited a significantly higher number of IELs per unit length compared to Groups A and C (*p* < 0.05), with Group D demonstrating the highest count. These findings suggest that the administration of *B. subtilis* 168 and RH can effectively enhance intestinal mucosa development and promote the maturation of intestinal structures in mice. Notably, *B. subtilis* RH appears to be more effective in stimulating the proliferation of IELs and modulating the immune response within the intestinal mucosa.

### 3.7. Mouse Immune Organ Index

**Figure 9:**
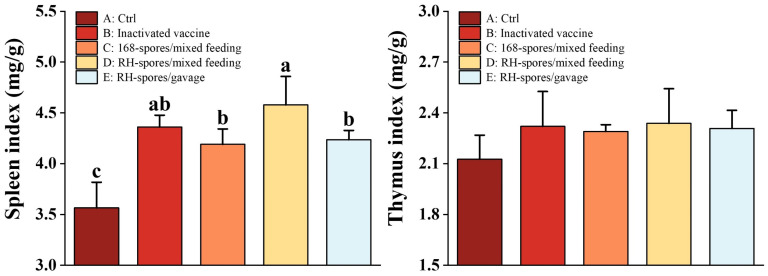
Organ indices of the thymus and spleen. Data are presented as mean ± SD, with a sample sized of *n* = 5. Statistical analysis was conducted using one-way ANOVA, followed by a Friedman test. Bars labeled with different lowercase letters indicate a statistically significant difference at the *p* < 0.05 level.

Figure 9 demonstrates that the spleen index was increased in all groups except for Group A. Notably, Group D exhibited a significantly higher spleen index than both Group C and Group E (*p* < 0.05). In contrast, no significant differences were found in the thymus index across all the groups (*p* > 0.05).

## 4. Discussion

In the realm of livestock and poultry production, probiotics are increasingly being adopted as a viable alternative to antibiotics [43]. *B. subtilis*, a probiotic species, has been acknowledged by the FDA as safe and is commonly incorporated into functional feed additives due to its robust probiotic characteristics and the convenience of its isolation and cultivation. This bacterium exhibits remarkable resistance to the harsh conditions within the gastrointestinal tract, enabling it to maintain potent biological activity in the digestive system [44,45]. It contributes to enhanced digestion by providing enzymes, antimicrobial peptides, and other advantageous metabolic byproducts, which collectively improve feed efficiency, growth performance, and the integrity of the gut barrier [46,47]. *B. subtilis* also plays a pivotal role in modulating the intestinal microbiota and the immune response, thereby warding off pathogenic microorganisms and preventing diseases [48,49,50]. In addition, *B. subtilis* is often employed as an engineering bacterium for the efficient expression of exogenous proteins. A technique for displaying proteins on the surface of *B. subtilis* spores was first proposed by Isticato et al. [51] in 2001. They successfully expressed a 459-amino-acid C-terminal fragment of the tetanus toxin (TTFC) on a spore surface, demonstrating its reactivity. With the elucidation of the *B. subtilis* genome and advances in proteomic research, this technique has been effectively utilized across various domains, including mucosal immunization, industrial enzyme production, environmental remediation, pharmaceutical development, and more [3]. Owing to its probiotic attributes and stability, *B. subtilis* holds promising advantages as a mucosal adjuvant for the delivery of antigenic proteins. Recombinant probiotics displaying the *HN* protein can elicit specific immune responses when used as mucosal vaccines, offering substantial potential in research and application areas. Our laboratory has successfully constructed several recombinant *B. subtilis* strains that display heterologous proteins, including *Salmonella* OmpC [52], Rotavirus VP8 [33], and Porcine circovirus type 2 Cap proteins [53]. In the current study, we excised a portion of the antigenic region from the *HN* protein and spliced it into smaller fragments following Chen’s methodology [32]. This approach preserved the native hemagglutinin–neuraminidase active sites of the *HN* protein, along with the majority of its antigenic sites and all the cysteine residues crucial for its tertiary structure. The resulting truncated *HN* protein is efficiently expressed in prokaryotic systems as a monomer, in contrast to the wild-type tetramer form. The prokaryotically expressed *HNJD* protein is readily recognized by NDV-specific antibodies, demonstrating robust reactivity. Immunofluorescence assays corroborated the accurate expression and correct anchoring of the *HNJD* protein to the spore surface during sporulation.

Upon ingestion by animals, *B. subtilis* spores can safely navigate through the stomach and germinate within the upper intestine, where they undergo a brief period of multiplication before sporulating again in the lower intestine [54,55]. Both spores and vegetative cells possess the capacity to interact with intestinal epithelial cells, thereby stimulating the proliferation of intestinal-associated lymphocytes and fortifying the mucosal immune system [56]. The mucosal immune system is pivotal for immune defense, acting as the body’s initial barrier against pathogenic invasion. In the context of preventive vaccination against Newcastle disease, eliciting an effective mucosal immune response can significantly diminish the rates of infection and morbidity, constituting the most efficacious strategy for the prevention and control of the disease [57]. In our investigation, we discovered that *B. subtilis* RH has the capacity to induce both systemic and local mucosal immune responses. It was observed that supplementing mice with *B. subtilis* RH resulted in elevated levels of serum IgG and intestinal sIgA. sIgA is the most abundant immunoglobulin produced by the intestinal mucosa, contributing to mucosal homeostasis by inhibiting pathogen adherence to the mucosal surface and playing a vital role in the early prevention of infection [58]. In contrast, commercial vaccines administered via intraperitoneal injection fail to stimulate the production of anti-NDV-specific sIgA in mice. This limitation is due to the fact that most commercial vaccines delivered through non-intestinal routes provide only partial protection against clinical infections and do not completely eliminate infection at the site of initial mucosa invasion. Chicks immunized with commercially inactivated vaccines often require mucosal immune adjuvants to elicit the effective production of specific mucosal antibodies, as indicated in previous studies [59,60]. HI and NA assays are used to measure antibody potency, reflecting their capacity to neutralize the virus and prevent infection. Through microneutralization and HI assays, we have shown that serum antibodies derived from mice vaccinated with RH-spores effectively neutralize NDV, preventing its cellular invasion. This suggests that antigen delivery via spores can effectively penetrate the selectively permeable mucus layer of the intestinal tract, leading to the generation of antibodies and the secretion of cytokines by associated lymphocytes [61,62], which contributes to combating disease infections. Gonçalves et al. [15] immunized zebrafish with spores displaying the *Vibrio* antigen OmpK, leading to a 50–90% increase in survival rates after infection with *Vibrio anguillarum* and *Vibrio parahaemolyticus*. Oh et al. [63] constructed a recombinant *B. subtilis* strain expressing the protective antigen (PA) of *Bacillus anthracis*, which successfully elicited a protective immune response in mice, with the level of protection varying according to the immunization method used. In our study, we observed that the immunogenic effect was dependent on the mode of administration, with the mixed feeding group showing superior performance over the intermittent gavage group in all measured parameters, including IgG and sIgA levels, as well as NA and HI titers. This could be attributed to the fact that dietary spores germinate in the jejunum and ileum, whereas spores and vegetative cells do not permanently colonize the intestine and are only in it for a brief duration [54,64]. Our findings suggest that continuous feeding immunization is more effective at inducing intestinal mucosal immunity and probiotic effects than high-dose gavage administered a limited number of times, aligning with previous research conducted in our laboratory. Additionally, gavage may induce stress and disrupt homeostasis in animals, indicating that intermittent gavage is not an effective or suitable immunization method.

In our study, *B. subtilis* RH modulated the expression levels of cytokines such as IL-1β, IL-6, IL-10, TNF-α, and IFN-γ in mouse ileal tissues. These cytokines are instrumental in orchestrating the Th1/Th2 pathway, which is essential for the regulation of both innate and adaptive immune responses in animals [65]. CD4^+^ T lymphocytes can differentiate into either Th1 or Th2 cell subsets. The elevated expression of IL-1β, TNF-α, and IFN-γ contributes to cellular immunity by fostering a converging Th1-type immune response. IFN-γ, acting as a pivotal activator of cellular immunity, stimulates CD8^+^ T lymphocytes and macrophages to eliminate foreign pathogens or infected cells. On the other hand, IL-6 and IL-10 respond to Th2 chemotaxis and also propel the proliferation of T and B cells, thereby enhancing the innate immune response [35]. Unlike many commercially inactivated vaccines that predominantly elicit Th2-type responses [66], *B. subtilis* RH is capable of promoting both cellular and innate immunity. The spores of the wild-type *B. subtilis* strain also upregulated cytokine expression, demonstrating their capacity to bolster the immune system. This observation aligns with the findings reported by LEE et al. [67].

Compared to conventional injectable vaccines, mucosal vaccines offer convenience, enhanced safety, and cost-effectiveness. Our investigation has demonstrated that *B. subtilis* RH can elicit a specific immune response when administered orally, highlighting its potential as an adjuvant for mucosal vaccines, which is particularly advantageous in the livestock and poultry industries. Measures such as gut histomorphometry and immune organ indices are frequently employed in research to assess gastrointestinal development and performance. The morphology of the small intestine directly mirrors the health of the gut, as well as its digestive and absorptive capabilities, which are critical for sustaining the intestinal immune system [68,69,70]. Longer villi correlate with epithelial cell proliferation and can stimulate cell mitosis, whereas shorter villi and deeper crypts are associated with nutrient malabsorption and diminished gastrointestinal function [71,72,73,74]. Our research has determined that dietary supplementation with *B. subtilis* RH results in an increase in the height of ileal villi and a decrease in crypt depth, thereby enhancing intestinal tissue structure in mice. In related findings, Dong et al. [75] found that *B. subtilis* BYS2 not only increased the height of avian duodenal and jejunal villi but also increased the villi-to-crypts rates, reduced viral load following NDV infection, and significantly improved survival rates. Jayaraman et al. [76] discovered that *B. subtilis* PB6 could replace antibiotic growth promoters (AGP) to prevent necrotizing enterocolitis while concurrently improving small intestine organization and the growth performance of broilers through mixed feeding. The intestinal mucosa’s epithelial layer is replete with IELs, which are crucial for preserving intestinal epithelial integrity and play a pivotal role in immune surveillance and cell-mediated mucosal immunity [56,77]. In our study, both strains of *B. subtilis* effectively stimulated the proliferation of IELs in the mouse ileum, thereby enhancing immunoprotection against virus challenges and potentially blocking the infection route of NDV. However, it remains uncertain if this effect is replicated with commercial vaccines. The organ index serves as an objective measure to assess the growth and development of immune organs and is a crucial parameter for evaluating the micro-ecological impacts of probiotics [78,79]. Our findings indicate that *B. subtilis* RH promotes splenic growth in mice, yet its influence on thymic development was not evident on day 42. This could be attributed to the fact that the thymus is among the first immune organs to develop and undergoes age-related involution [80], culminating in no significant disparity between the experimental groups. Furthermore, *B. subtilis* RH has been utilized as a genetically engineered probiotic for animal feed at the recommended dosage (1.0–2.0 × 10^6^ CFU/g), which is consistent with that of most probiotics [81,82]. This approach ensures that it functions as an immune adjuvant while eliciting an effective immune response. In a related study by Pham et al. [83], when the conventional dose (1.0 × 10^6^ CFU/g) of CotB-VP28 spores in feed pellets was increased by 50-fold (5.0 × 10^7^ CFU/g) or 1000-fold (1.0 × 10^9^ CFU/g) for black tiger shrimps, the enhancement in protection against spot syndrome virus infection was marginally increased by only 2.5% and 7.5%, respectively. Thus, routine dosages of spore supplementation can provide effective immune protection, and higher concentrations are not only unnecessary but also impractical in the poultry industry due to cost implications. Nonetheless, we posit that the optimal concentration of various recombinant *B. subtilis* strains may differ depending on the vectors used, the expression methods, and efficiency. Future poultry experiments could explore the immunoprotective effects across different dosages. In summary, these findings indicate that *B. subtilis* RH, when used as a feed additive, significantly enhances the development of intestinal and immune organs in animals, promotes the proliferation of immune-related lymphocytes, and shows promise as a mucosal immune adjuvant.

Although *B. subtilis* RH was capable of inducing an effective specific immune response, the IgG levels and NA titers were not as high as those achieved with commercial vaccines. One plausible explanation for this discrepancy could be the absence of a linker peptide, which might impact the expression of exogenous proteins. The inclusion of a linker peptide can form a stable helical structure that bridges the anchor protein and the target protein, alleviating rigidity issues and enhancing their stability and expression. However, the direct fusion of anchor proteins may lead to undesirable outcomes such as incorrect folding, low yields, and compromised biological activity [84]. Hinc et al. [85] compared the expression efficiency of fusion proteins in *cotZ*-UreA versus *cotB*-GGGEAAAKGGGG-UreA, finding that the latter, which incorporates flexible linker peptides, expressed up to 100-fold more recombinant proteins on a spore than the former. Although various types of linker peptides have been designed over time [86,87], the ideal configuration remains elusive. The selection of different anchor proteins [8,85] and ligation strategies [88,89] can also significantly enhance the presentation efficiency of exogenous proteins. Determining how to improve the display efficiency of the *HNJD* protein on the spore surface may be a focus of our future research endeavors. Looking ahead, omics technologies should be harnessed to uncover the precise mechanisms through which *B. subtilis* RH induces immune responses and exerts its probiotic effects.

## 5. Conclusions

In this study, we developed a recombinant *B. subtilis* RH strain capable of expressing the NDV *HN* protein on the surface of its spores. This innovation induced both mucosal and systemic immune responses in mice. Additionally, the recombinant *B. subtilis* enhanced the expression of cytokines in the ileum, fostered the development of immune organs and the intestinal tract, and stimulated the proliferation of intestinal-associated lymphocytes. As such, it also serves as a mucosal immune adjuvant, positioning it as a potential novel probiotic strain engineered to combat NDV. The evaluation of its immunogenicity in mice conducted herein lays the groundwork for future challenge studies in poultry models.

## Data Availability

Data are contained within the article.

## Supplementary Materials

The following supporting information can be downloaded at https://www.mdpi.com/article/10.3390/microorganisms12030439/s1. The supplementary files provided include Table S1, which lists the trains, vaccines, cell lines, plasmids, and primer sequences used; Table S2, detailing the primer sequences for real-time quantitative PCR; and Table S3, presenting the *HNJD* gene sequences along with the optimized sites.
