# Supplementary material for: Evaluation of the Immunity Responses in Mice to Recombinant Bacillus subtilis Displaying Newcastle Disease Virus HN Protein Truncations
Source: Microorganisms. 2024 Feb 21;12(3):439. doi: 10.3390/microorganisms12030439 (PMC10972176; doi:10.3390/microorganisms12030439)
Supplement: Supplementary file 1 [file microorganisms-12-00439-s001.zip › microorganisms-2813318-supplementary.pdf]

Supplementary materials for

Evaluation of Immune Responses in mice to Recombinant *Bacillus subtilis* Displaying Newcastle Disease Virus *HN* Protein Truncations

Jianzhen Li [1, 2, †], Miao Yang [3, †], Bin Chen [1], Zhenhua Wang [2], Yuheng Cao [1], Yang Yang [1], Mengwei Zhang [1], Dongmei Zhang [1], Xueqin Ni [1], Yan Zeng [1, *] and Kangcheng Pan [1, *]

The file includes:

Table S1. List of strains, vaccines, cell lines, plasmids, and primer sequences.

Table S2. Sequences of primers used for real-time quantitative PCR.

Table S3. *HNJD* gene sequences and sites of optimization.

Table S1. List of strains, vaccines, cell lines, plasmids, and primer sequences.

Strains, Vaccines, Cell lines，Plasmids, and Primer Sequences	Description	Source or Restriction Site
**Strains**		
Escherichia coli (*E.coli*) DH5α and BL21	Competent cell strains	TIANGEN, Beijing, China
B. subtilis 168	Genbank accession: AL009126.3	Our lab
B. subtilis RH	*B. subtilis* 168 *amyE*:: *cotB-HNJD*	This work
**Vaccines**		
Newcastle disease vaccine	Live (Strain LaSota)	HARVAC, Harbin, China
	Live (Strain CS2)	EBVAC, Hangzhou, China
	Inactivated (Strain La Sota)	Yebio, Qingdao, China
**Cell lines**		
Chicken embryo fibroblast (CEF)	UMNSAH/DF-1	SUNNCELL, Wuhan, China
**Plasmids**		
pUCm-T	T-A cloning vector	Sangon, Shanghai, China
pET-32a-*HNJD*	Prokaryotic expression vector	This work
pDG364	*E. coli-B. subtilis* shuttle vector	Our lab
pDG364-*cotB*	pDG364 derivative carrying *cotB* gene	Our previous work
pDG364-*cotB-HNJD*	pDG364 derivative carrying the fusion *cotB-HNJD* gene	This work
**Primer Sequences**		
HNJD-F1	5’-CGCGGATCCATGGTGATATACAAGCGATACAATGAC-3’	*BamH* I
HNJD-R1	5’-CCGGAATTCTTAAGCAATGCTGAGACAATAGGT-3’	*EcoR* I
HNJD-F2	5’-CCCAAGCTTATGGTGATATACAAGCGATACAATGAC-3’	*Hind* III
HNJD-R2	5’-CCGGAATTCTTAAGCAATGCTGAGACAATAGGT-3’	*EcoR* I
cotB-F	5’-CGGGATCCACGGATTAGGCCGTTTGTCC-3’	*BamH* I
cotB-R	5’-GGGAAGCTTGGATGATTGATCATCTGAAG-3’	*Hind* III
amyE-F	5’-CCAATGAGGTTAAGAGTATTCC-3’	Null
amyE-R	5’-CGAGAAGCTATCACCGCCCAGC-3’	Null

Table S2. Sequences of primers used for real-time quantitative PCR.

Gene	Primer Sequences (5'-3')		Accession Number
β-actin	F: GCTCTTTTCCAGCCTTCCTT	R: GATGTCAACGTCACACTT	NM_007393.5
IL-1β	F: ATGAAAGACGGCACCCCAC	R: GCTTGTGCTCTGCTTGTGAG	NM_008361.3
IL-6	F: TGCAAGAGACTTCCATCCAGT	R: GTGAAGTAGGGAAGGCCG	NM_031168.1
IL-10	F: GGTTGCCAAGCCTTATCGGA	R: ACCTGCTCCACTGCCTTGCT	NM_010548.2
IFN-γ	F: TCAAGTGGCATAGATGTGGAAGAA	R: TGGCTCTGCAGGATTTTCATG	NM_008337.4
TNF-α	F: ACGGCATGGATCTCAAAGAC	R: AGATAGCAAATCGGCTGACG	NM_001278601.1

Table S3. *HNJD* gene sequences and sites of optimization.

Original *HNJD*	Optimized *HNJD*
*ATGGTGATATACAAGCGATACAATGACACATG	ATGGTGATATACAAGCGATACAATGACACATG
CCCAGATGAGCAAGACTACCAGATTCGAATGG	CCCAGATGAGCAAGACTACCAGATTCGAATGG
CCAAGTCTTCGTATAAGCCTGGACGGTTTGGT	CCAAGTCTTCGTATAAGCCTGGACGGTTTGGT
GGGAAACGCATACAGCAGGCTATCTTATCTATC	GGGAAACGCATACAGCAGGCTATCTTATCTATC
AAGGTGTCAACATCCTTAGGCGAAGACCCGGT	AAGGTGTCAACATCCTTAGGCGAAGACCCGGT
ACTGACTGTACCGCCCAACACAGTCACACTCA	ACTGACTGTACCGCCCAACACAGTCACACTCA
TGGGGGCCGAAGGCAGAATTCTCACAGTAGG	TGGGGGCCGAAGGCAGGATTCTCACAGTAGG
GACATCTCATTTCTTGTATCAACGAGGGTCATC	GACATCTCATTTCTTGTATCAACGAGGGTCATC
ATACTTCTCTCCCGCGTTATTATATCCTATGACA	ATACTTCTCTCCCGCGTTATTATATCCTATGACA
GTCAGCAACAAAACAGCCACTCTTCATAGTCC	GTCAGCAACAAAACAGCCACTCTTCATAGTCC
TTATACATTCAATGCCTTCACTCGGCCAGGTAG	TTATACATTCAATGCCTTCACTCGGCCAGGTAG
TATCCCTTGCCAGGCTTCAGCAAGATGCCCCA	TATCCCTTGCCAGGCTTCAGCAAGATGCCCCA
ACTCGTGTGTTACTGGAGTCTATACAGATCCAT	ACTCGTGTGTTACTGGAGTCTATACAGATCCAT
ATCCCCTAATCTTCTATAGAAACCACACCTTGC	ATCCCCTAATCTTCTATAGAAACCACACCTTGC
GAGGGGTATTCGGGACAATGCTTGATGGTGTA	GAGGGGTATTCGGGACAATGCTTGATGGTGTA
CAAGCAAGACTTAACCCTGCGTCTGCAGTATT	CAAGCAAGACTTAACCCTGCGTCTGCAGTATT
CGATAGCACATCCCGCAGTCGCATTACACGAG	CGATAGCACATCCCGCAGTCGCATTACACGAG
TGAGTTCAAGCAGTACCAAAGCAGCATACACA	TGAGTTCAAGCAGTACCAAAGCAGCATACACA
ACATCAACTTGTTTTAAAGTGGTCAAGACTAA	ACATCAACTTGTTTTAAAGTGGTCAAGACTAA
TAAGACCTATTGTCTCAGCATTGCTTAA	TAAGACCTATTGTCTCAGCATTGCTTAA

*The yellow-highlighted sequences indicate the optimized sites, while the red-highlighted sequences denote the start codons.
